# Effect of e-Health Literacy on COVID-19 Infection-Preventive Behaviors of Undergraduate Students Majoring in Healthcare

**DOI:** 10.3390/healthcare9050573

**Published:** 2021-05-12

**Authors:** Kyung Jin Hong, Noo Lee Park, Soo Yeon Heo, Seo Hyun Jung, Ye Been Lee, Ji Hoon Hwang

**Affiliations:** Department of Nursing, Semyung University, Jecheon 27136, Korea; 2018124021@semyung.ac.kr (N.L.P.); cindyi9@semyung.ac.kr (S.Y.H.); seo4436@semyung.ac.kr (S.H.J.); 2018124055@semyung.ac.kr (Y.B.L.); pt7512@semyung.ac.kr (J.H.H.)

**Keywords:** e-Health literacy, COVID-19, preventive behavior, undergraduate students

## Abstract

This study examined and verified the level of e-health literacy (e-HL) and infection preventive behaviors related to COVID-19 among undergraduate students majoring in healthcare. An online survey was conducted with 274 university students majoring in nursing, clinical pathology, and occupational therapy in South Korea. The e-HL consisted of functional, communicational, and critical literacy, and preventive behaviors were based on the Prevention Guideline on Droplet Infection. The mean score for e-HL was 3.62, with nursing students obtaining the highest scores. The overall e-HL score and the scores on its three sub-dimensions were related to infection-preventive behaviors. Moreover, e-HL affected infection-preventive behaviors (*p* < 0.001). Findings from this study highlight the necessity of education for improving the e-HL of undergraduate students majoring in healthcare to strengthen infection-preventive behaviors and protect patients from infectious diseases.

## 1. Introduction

The development of various social media platforms has enabled physicians and healthcare workers as well as the public to access health-related information quickly [1]. Consumers can search necessary health information via the Internet and use it for disease management, promotion of health status, and choice of healthcare facilities. Therefore, the importance of the Internet as a source of health-related information has increased [2]. The recent widespread distribution of mobile devices has also produced significant changes to the media ecosystem. People are now able to actively utilize the Internet, including social media, as an information source in addition to conventional mass media, such as newspapers and broadcasts. This has diversified the ways to access healthcare information [3].

The outbreak of coronavirus-19 (COVID-19), which began in 2019, led to serious global problems. On 30 January 2020, the World Health Organization (WHO) declared the new disease to be a Public Health Emergency of International Concern [4] and declared it a pandemic on 11 March 2020 [5]. Thus, to prevent the spread of COVID-19, it became crucial to notify the public about disease-related information and promote and recommend infection-preventive behaviors, including hand hygiene and wearing a face mask [6]. Information about COVID-19 has been broadly distributed via the Internet since the Internet facilitates the rapid spread of information. Among the various information sources, the Internet is the most frequently used by the general public to explore highly uncertain risk factors, such as those regarding new infectious diseases such as COVID-19 [7]. In fact, a study on the route of acquiring information related to COVID-19 in six different countries revealed that most people used social media, search engines, video content websites, and message applications to obtain COVID-19-related news and information [8]. However, many people reported difficulty in judging whether they could trust various media channels [9] and handle health-related information [10]. Since the information found on mass media and the Internet may be relayed to others through human information processing strategies with an indirect impact on risk awareness and preventive behaviors [11], it is necessary to have the ability to critically analyze and select essential information from a vast array of data.

The ability to search for appropriate healthcare information on the Internet and use it correctly is known as e-health literacy (e-HL). Hence, e-HL is the ability to use a computer, search information, understand healthcare information, and apply the findings to a given circumstance appropriately [12]. It combines health literacy with digital literacy and indicates the ability to screen health information available online and to interpret and assess the information obtained [12]. e-HL is a key factor affecting infection-preventive behaviors related to COVID-19 [13] and psychological status [14]. Its importance has grown continuously with the increasing amount of information available online.

In a study investigating Internet use by different age groups in South Korea in 2019, 99.9% of individuals in their 20s used the web, mobile Internet, and smartphones for communication (95.4%) and leisure (94%), followed by the acquisition of information (94%) [15]. This suggests that the use of media and the consequent acquisition of information have become highly natural. While individuals in their 20s are generally in their healthiest and most physically active time of life, they are therefore more easily exposed to the risk of infection [16]. A survey conducted in South Korea regarding public awareness of self-quarantine due to COVID-19 revealed that the level of awareness and adherence regarding infection control was low in individuals in their 20s, and since their subjective health status was high, their adherence to government guidelines such as refrain from using public facilities was reduced [16]. This implies the need to investigate the infection-preventive behaviors of undergraduate students in their 20s, especially those majoring in healthcare who, as healthcare professionals, would acquire the responsibility to prevent the spread of pathogenic agents as well as viruses through the accurate and diligent performance of infection control and preventive behaviors.

Previous research highlighted the importance of e-HL in nurses so that they can correct patients’ misconceptions regarding their illness due to incorrect interpretation of information found online while guiding them to use such information correctly [17,18,19]. Along with nurses, nursing students, as well as undergraduate students majoring in all other healthcare fields, should have a critical view of the healthcare information that can be obtained on the Internet, as they would play the role of propagating correct healthcare information to patients in the future, as healthcare professionals or technicians. Furthermore, to prevent the spread of infection, medical institutions should urgently conduct studies to enhance infection-preventive behaviors. The COVID-19 hospital-acquired rate was 12–15%, and the necessity of compliance with all procedures for preventing the spread of hospital-associated COVID-19 infection and for healthcare personnel is being emphasized [20]. This study investigated the level of e-HL among undergraduate students majoring in healthcare and determine the effect of e-HL on such behaviors.

The current study aimed (1) to report the level of e-HL and infection-preventive behaviors among students majoring in healthcare, (2) to examine the differences in e-HL and infection-preventive behaviors according to students’ general characteristics, and (3) to verify the effect of e-HL on infection-preventive behaviors.

## 2. Materials and Methods

### 2.1. Study Design

This study adopted a descriptive, questionnaire-based cross-sectional approach.

### 2.2. Setting and Participants

The participants were undergraduate students majoring in healthcare (nursing, clinical pathology, and occupational therapy) at a university in South Korea who had submitted their consent for participation. G* power 3.1.9.7 was used to calculate the number of participants required for this study. The sample size was estimated at a significance level of 0.05, the effect size of 0.25, and statistical power of 0.95, indicating that 210 participants were required. Considering the dropout rate, data were collected from a total of 279 participants. After excluding five participants due to incomplete responses, the data of 274 participants were analyzed.

### 2.3. Data Collection and Procedure

To recruit undergraduate students majoring in nursing, clinical pathology, and occupational therapy at the aforementioned university, a notice was posted on the boards of the respective departments following the approval of the Institutional Review Board. The notice contained the title, detailed explanations of the purpose, the survey procedures and duration, and the compensation provided, along with the URL of the online survey, so that interested students could visit the page describing the study and complete the consent form. To participate, the students had to click the box “I consent to participation”, which directed them to the online survey. The survey was conducted from 14–16 September 2020.

### 2.4. Measures

#### 2.4.1. General Characteristics of Participants

The general characteristics of the participants included gender, major, and grade at the university. A five-point Likert scale was used to assess their health status and health concerns. Regarding health status (1 = very unhealthy, 5 = very healthy), the respondents who rated this as 1, 2, or 3 were grouped as *poor or moderate*, and those who rated it 4 or 5 were grouped as *healthy*. Regarding health concerns (1 = very low, 5 = very high), the respondents who rated this as 1, 2, or 3 were grouped as *low or moderate*, and those who rated it as 4 or 5 were grouped as *high*. Regarding health management time, the participants were divided into the following groups: <1 h, 1–<4 h, and ≥4 h.

#### 2.4.2. e-Health Literacy

The level of e-HL was measured using the e-HL tool developed by Lee (2018) [21]. The tool comprised a questionnaire of 31 items regarding functional e-HL (8 items), communicative e-HL (11 items), and critical e-HL (12 items). Functional e-HL refers to the ability to read and write health-related information online. The items included “I can search for the necessary health information online” and “I can locate the necessary health information rapidly online.” Cronbach’s α was 0.90 in a previous study [21] and 0.91 in the current study.

Communicative e-HL refers to the ability to extract healthcare information online and interpret and relay the obtained information via various forms of intercommunication. The items included “I can talk about my health status with another individual online”, “I can exchange opinions about which hospital to visit”, and “I can communicate with another individual online to determine my health status”, along with eight other items. Cronbach’s α was 0.90 in a previous study [21] and 0.92 in the current study.

Critical e-HL is the advanced cognitive ability to critically analyze healthcare information online and to adjust and apply it as appropriate to one’s health status. The questions for critical e-HL included items such as “I can determine whether the health information found online is credible”, “I can decide whether the health information found online applies to my health status”, and “I can collect and determine specific health information online”, along with nine other items. Cronbach’s α was 0.92 in a previous study [21] and 0.92 in the current study. Each item was rated on a five-point Likert scale (1 = very unlikely, 5 = very likely), where a higher score indicated that the respondent had a higher level of e-HL and less difficulty in using health-related information found online [21].

#### 2.4.3. Infection-Preventive Behaviors

Infection-preventive behaviors were examined using a tool developed by Lee et al. (2016) based on the Prevention Guideline on Droplet Infection [22]. It contained six items: “Do not touch your eyes, nose, or mouth with unwashed hands”, “Cover your mouth with a tissue or handkerchief when you sneeze or cough”, “Avoid contact with someone showing respiratory symptoms or fever”, “Wear a mask outdoors”, “Avoid crowded places”, and “Refrain from visiting hospitals or medical institutions.” A five-point Likert scale was used, with a higher mean score indicating a higher level of infection-preventive behaviors. The measure’s reliability was reported as Cronbach’s α = 0.90 in a previous study [22] and 0.63 in the current study.

### 2.5. Analysis

Descriptive analysis methods, including frequency, percentage, mean, and standard deviation, were used to analyze the participants’ general characteristics and the level of e-HL and infection-preventive behaviors. To examine the differences in the level of e-HL and infection-preventive behaviors according to their general characteristics, a *t*-test and ANOVA were performed, while the Scheffe test was applied as a post-hoc test. The correlation between e-HL and infection-preventive behaviors was analyzed using Pearson correlation, and the factors affecting these behaviors were analyzed using multiple regression analysis.

## 3. Results

### 3.1. Characteristics of Participants and the Level of e-HL and Infection-Preventive Behaviors

Table 1 displays participants’ general characteristics and the level of e-HL and infection-preventive behaviors. About 237 (86.5%) participants were female and 138 (50.4%) were majoring in nursing, followed by occupational therapy and clinical pathology. For each grade, the largest number of students belonged to Grade 2 (90 students; 32.8%), followed by Grades 1, 3, and 4. Regarding their current health status and health concerns, the number of students was larger in the healthy and high groups than in the poor/low or moderate groups. The daily mean health management time was < 1 h for 122 students (44.5%), 1–<4 h for 85 students (31.0%), and ≥4 h for 67 students (24.4%). The mean e-HL score was 3.62. For each sub-dimension, the mean scores for functional, communicative, and critical e-HL were 3.98, 3.41, and 3.58, respectively. The mean score for infection-preventive behavior was 4.22. The highest mean score was 4.91 for “wearing a mask outdoors”, whereas the lowest mean score was 3.46 for “not touching eyes or mouth with unwashed hands.”

### 3.2. e-HL and Infection-Preventive Behaviors according to the General Characteristics

Table 2 presents the differences in e-HL and infection-preventive behaviors according to the general characteristics of the participants. The e-HL mean score of students majoring in nursing was higher than that of students majoring in clinical pathology and occupational therapy (F = 6.54, *p* = 0.002). The mean scores of e-HL and infection-preventive behaviors of students who perceived their health status as healthy were higher than those who perceived their health status as poor or moderate (*t* = −2.71, *p*= 0.007; *t* = –3.23, *p* = 0.001). Moreover, the mean scores of e-HL and infection-preventive behaviors of students whose health concerns were high were significantly higher than those with low or moderate health concerns (*t* = –4.71, *p* < 0.001; *t* = –3.27, *p* = 0.001).

### 3.3. Correlations between e-HL and Infection-Preventive Behaviors

Table 3 reports the correlations between e-HL and infection-preventive behaviors. Overall health literacy was significantly positively related to functional (r = 0.77, *p* < 0.001), communicative (r = 0.86, *p* < 0.001), and critical e-HL (r = 0.88, *p* < 0.001). Moreover, the overall e-HL score and all the e-HL sub-dimensions were positively related to infection-preventive behaviors.

### 3.4. Regression Results on Infection-Preventive Behaviors

Table 4 shows the factors affecting infection-preventive behaviors. The overall e-HL score was positively associated with infection-preventive behaviors (*p* < 0.001). The infection-preventive behavior score of third-year students was lower than that of first-year students.

## 4. Discussion

This study investigated the level of e-HL and COVID-19 infection-preventive behaviors of undergraduate students majoring in healthcare, as well as the effect of e-HL on such behaviors. Furthermore, this study discussed the necessity of providing an e-HL enhancing education program to prevent hospital-associated infection among healthcare personnel. The mean score of e-HL was 3.62, which was higher than the 3.51 reported in a previous study using the same tool targeting general undergraduate students [23]. This was consistent with the results of other studies where the level of e-HL was higher among undergraduate students majoring in healthcare since they spent more time studying and being exposed to health-related information compared to those of other majors [18,23,24,25]. Regarding the level of e-HL according to the major, the mean score was 3.75 for nursing, which was higher than that of clinical pathology or occupational therapy. This may be attributed to the fact that students majoring in nursing obtained healthcare knowledge in an integrated way regarding the promotion of human well-being and health.

For infection-preventive behaviors, “Wear a mask outdoors” showed the highest mean score (4.91), while “Do not touch your eyes, nose, or mouth with unwashed hands” showed the lowest score (3.46). In South Korea, public awareness of wearing a mask in public places is relatively high due to the continuous emphasis in the media. Additionally, it has been regarded as the basic rule of COVID-19 prevention and has been made mandatory by the government since November 2020. This is presumed to be the reason behind this factor having the highest score among the preventive behaviors. Furthermore, the participants in this study refrained from going out unnecessarily, avoided crowded places, and minimized visits to medical institutions. High adherence to these measures may because they are part of the official guidelines for preventing COVID-19 conveyed by the Ministry of Health and Welfare [26]. Nonetheless, adherence to preventive behaviors, such as not touching the eyes, nose, or mouth with unwashed hands, was found to be low as they are habits that may recur unconsciously.

The level of e-HL according to the health status was significantly higher in the healthy group than in the poor or moderate group. This is consistent with the high e-HL in relation to high subjective health status in previous studies [27]. Having a cross-sectional design, the current study did not indicate a clear cause-effect relationship. Low health status may have been due to low e-HL, which prevented adequate search and use of healthcare information. On the other hand, low health status may have led to a reduced ability to search for various healthcare information and critically analyze and apply it to improve health. Further studies should clearly identify the cause-effect relationship between health status and e-HL and discuss ways to use e-HL to improve health. The level of e-HL according to health concerns also showed significant intergroup differences; the high group showed higher e-HL and infection-preventive behaviors than the low or moderate group. Consistent with a previous study [22], a higher level of health concern led individuals to take more care of their health, thereby improving their e-HL.

A positive correlation was observed between overall e-HL and infection-preventive behaviors (r = 0.29, *p* < 0.001). The result of multiple regression analysis of the factors affecting such behaviors indicated that a high level of e-HL led to a high adherence to infection-preventive behaviors. This is consistent with a study on undergraduate students where e-HL correlated with getting vaccinations, and a study conducted in China on nursing students where e-HL correlated with COVID-19 infection-control behaviors [28,29]. e-HL indicates the ability to use the Internet to search, understand, and apply the necessary healthcare information with respect to health-related behaviors. A higher level of e-HL indicates a higher probability that the individual can obtain knowledge of infectious diseases, such as COVID-19, search relevant facts about the infection route and preventive behaviors using the Internet, and locate the necessary information and apply it in practice [30].

As can be seen in the case of COVID-19, adherence to preventive behaviors against an infectious disease would bring about consequences related to one’s own and others’ health, with a wide scope of effects on national policies and economics. Thus, it is necessary to encourage people to adhere to infection-preventive behaviors, and one method to do so is to enhance their level of e-HL. This highlights the need to formulate education programs that enhance the e-HL levels of undergraduate students. The institutions that provide information related to infectious diseases, such as the Ministry of Health and Welfare, should generate data in accordance with the existing level of e-HL and take a suitable route to deliver this information. Since the students majoring in nursing, clinical pathology, and occupational therapy, in particular, are going to be responsible for the prevention of infectious diseases in the future, their preparedness for patient interaction must be assessed properly [31], while each course in their respective departments should include education to enhance e-HL. Several studies have examined the effects of e-HL intervention among older adults. The intervention program’s goals included learning how to explore a website, find answers to health questions of personal interest, and evaluate the reliability of healthcare information websites [32]. With these references, intervention programs should be developed to enhance their ability to communicate with others about health problems and preventive strategies, and critically evaluate the health-related information available online to identify valuable and reliable information.

The present findings confirmed the crucial role of e-HL; notably, this study is significant in that the level of e-HL and infection-preventive behaviors were investigated among undergraduate students majoring in healthcare who will be healthcare professionals in the future. Recently, the importance of patient safety has been increasingly highlighted to emphasize the need for education to enhance the level of e-HL and infection-preventive behaviors among undergraduate students. Particularly, since this study verified the effects of e-HL on infection-preventive behaviors, further studies should search for ways to enhance the level of e-HL using a multidisciplinary approach.

This study had several limitations. As a cross-sectional study, it did not indicate a clear causal relationship. Additionally, since the participants were students from a single university and most were women, generalization of the results may be difficult. In the future, surveys should be conducted among individuals with varied characteristics. Furthermore, there may be differences between the results and the actual adherence to infection-preventive behaviors in practice, since the study assessed infection-preventive behaviors based on participants’ responses to a questionnaire. Therefore, future studies should develop a tool to measure the participants’ preventive behaviors more accurately. Further studies are needed to examine the association between e-HL and the actual infection rate. The tool’s model should also reflect numerous variables, including the level of health-related knowledge.

## 5. Conclusions

The current study examined the level of e-HL and infection-preventive behaviors and verified the factors affecting infection-preventive behaviors among undergraduate students majoring in healthcare. The level of e-HL among undergraduate students was related to their infection-preventive behaviors. Therefore, the need for the development of education programs to enhance the e-HL level were discussed. Healthcare-associated infection control is a fundamental role of all healthcare workers in patient safety. High levels of e-HL among future students majoring in healthcare would help prevent healthcare-associated infection in other people, especially patients. Moreover, further research should examine the factors affecting e-HL and the effectiveness of education programs in enhancing e-HL. Public institutions should provide information considering the public’s e-HL levels to prevent the transmission of infectious diseases.

## Figures and Tables

**Table 1:** Characteristics of participants and the level of e-HL and infection-preventive behaviors.

Variables	Categories	N (%) or M ± SD	Range (Min-Max)
Gender	Male	37 (13.5)	
	Female	237 (86.5)	
Major	Nursing	138 (50.4)	
	Clinical pathology	43 (15.7)	
	Occupational therapy	93 (33.9)	
Grade	1	79 (28.8)	
	2	90 (32.8)	
	3	60 (21.9)	
	4	45 (16.4)	
Health status	Poor or moderate	100 (36.4)	
	Healthy	174 (63.5)	
Health concerns	Low or moderate	99 (36.1)	
	High	175 (63.8)	
Health management time (h)	<1	122 (44.5)	
	1–<4	85 (31.0)	
	≥4	67 (24.4)	
e-Health literacy	Overall	3.62 ± 0.60	1–5 (2.06–5.00)
	Functional	3.98 ± 0.66	1–5 (1.13–5.00)
	Communicative	3.41 ± 0.80	1–5 (1.09–5.00)
	Critical	3.58 ± 0.66	1–5 (1.92–5.00)
Preventive behaviors	Overall	4.22 ± 0.51	1–5 (2.50–5.00)
	Not touching eyes or mouth with unwashed hands	3.46 ± 1.04	1–5 (1–5)
	Covering mouth when sneezing or coughing	3.71 ± 1.13	1–5 (1–5)
	Avoiding someone showing respiratory symptoms or fever	4.46 ± 0.80	1–5 (1–5)
	Wearing a mask outdoors	4.91 ± 0.36	1–5 (3–5)
	Avoiding crowded places	4.41 ± 0.78	1–5 (2–5)
	Refraining from visiting hospitals	4.37 ± 0.87	1–5 (1–5)

**Table 2:** Differences of e-HL and infection-preventive behaviors according to the participants’ general characteristics.

Variables	Categories	e-Health Literacy		Preventive Behavior	
		M ± SD	*t* or F	M ± SD	*t* or F
Gender	Male	3.74 ± 0.62	1.29 (0.199)	4.20 ± 0.55	−0.22 (0.824)
	Female	3.61 ± 0.60		4.22 ± 0.51	
Major	Nursing	3.75 ± 0.57	6.54 (0.002) a > b, c	4.29 ± 0.50	2.56 (0.079)
	Clinical pathology	3.44 ± 0.47		4.12 ± 0.54	
	Occupational therapy	3.52 ± 0.67		4.17 ± 0.52	
Grade	1	3.59 ± 0.61	1.19 (0.313)	4.30 ± 0.52	2.46 (0.063)
	2	3.63 ± 0.57		4.24 ± 0.48	
	3	3.55 ± 0.67		4.07 ± 0.55	
	4	3.76 ± 0.55		4.24 ± 0.50	
Health status	Poor or moderate	3.50 ± 0.56	−2.71 (0.007)	4.09 ± 0.54	−3.23 (0.001)
	Healthy	3.70 ± 0.61		4.30 ± 0.48	
Health concerns	Low or moderate	3.40 ± 0.58	−4.71 (<0.001)	4.09 ± 0.55	−3.27 (0.001)
	High	3.75 ± 0.58		4.30 ± 0.48	
Health management time (h)	<1	3.54 ± 0.60	2.52 (0.082)	4.16 ± 0.54	2.55 (0.080)
	1–<4	3.64 ± 0.60		4.21 ± 0.47	
	≥4	3.75 ± 0.60		4.34 ± 0.51	

**Table 3:** Correlations among e-HL and infection-preventive behaviors.

Variables	Functional e-HL	Communicative e-HL	Critical e-HL	Overall e-HL	Preventive Behaviors
Functional e-HL	1.00				
Communicative e-HL	0.47 (<0.001)	1.00			
Critical e-HL	0.62 (<0.001)	0.60 (<0.001)	1.00		
Overall e-HL	0.77 (<0.001)	0.86 (<0.001)	0.88 (<0.001)	1.00	
Preventive behaviors	0.24 (0.001)	0.19 (0.001)	0.30 (<0.001)	0.29 (<0.001)	1.00

HL: health literacy.

**Table 4:** Regression results in infection-preventive behaviors.

Variables	Categories	Beta	SE	*t*	*p*
e-Health literacy		0.23	0.05	3.81	<0.001
Gender (vs. male)	Female	0.08	0.09	1.37	0.173
Major (vs. nursing)	Clinical pathology	−0.01	0.09	−0.20	0.840
	Occupational therapy	−0.03	0.07	−0.41	0.685
Grade (vs. 1)	2	−0.05	0.08	−0.74	0.457
	3	−0.17	0.09	−2.41	0.016
	4	−0.07	0.09	−1.09	0.277
Health status (vs. poor or moderate)	Healthy	0.11	0.06	1.87	0.062
Health concerns (vs. low or moderate)	High	0.09	0.07	1.42	0.157
Health management time (h, vs. <1)	1–<4	−0.02	0.07	−0.24	0.810
	≥4	0.07	0.08	1.07	0.284

R^2^ = 0.14, Adj. R^2^ = 0.10, F = 4.01, *p* < 0.001

## Data Availability

The data presented in this study are available upon request.
